# Effect of Conventional and Ultrasound-Assisted Extraction Conditions on the Physicochemical Properties, Phytochemical Content, Antioxidant Activity and Functional Properties of Alfalfa Protein Concentrates

**DOI:** 10.3390/foods14244309

**Published:** 2025-12-14

**Authors:** Angela Gurev, Viorica Bulgaru, Iana Ciugureanu, Natalia Netreba, Veronica Dragancea, Irina Dianu, Iuliana Sandu, Mihail Mazur, Tatiana Mitina, Nadejda Bandarenco, Aliona Ghendov-Mosanu

**Affiliations:** 1 Faculty of Food Technology, Technical University of Moldova, 9/9 Studentilor St., MD-2045 Chisinau, Moldova; angela.gurev@chim.utm.md (A.G.); iana.ciugureanu@doctorat.utm.md (I.C.); natalia.netreba@tpa.utm.md (N.N.); veronica.dragancea@chim.utm.md (V.D.); irina.dianu@doctorat.utm.md (I.D.); iulia.sandu@tpa.utm.md (I.S.); mihaimazur9@gmail.com (M.M.); aliona.mosanu@tpa.utm.md (A.G.-M.); 2 “ILAS”AL Testing Laboratory, Institute of Chemistry, Moldova State University, 60 Alexei Mateevici St., MD-2009 Chisinau, Moldova; mitina.tatiana@sti.usm.md (T.M.); bandarenco.nadejda@sti.usm.md (N.B.)

**Keywords:** alfalfa, protein concentrate, ultrasound-assisted extraction, protein digestibility, antioxidant activity, bioactive compounds, functional proprieties

## Abstract

Alfalfa (*Medicago sativa* L.) is an underutilized source of phytonutrients and easily digestible protein, containing all essential amino acids, highlighting its potential for food applications. This study aimed to produce alfalfa protein concentrates (APC) from frozen aerial parts and evaluate how conventional extraction and ultrasound-assisted extraction (UAE) affect the extraction yield, physicochemical properties, functional attributes, color parameters, phytochemical composition and antioxidant activity. The influence of extraction pH and the type of acid used for isoelectric precipitation was also evaluated. Paired *t*-tests (*p* ≤ 0.05) showed that UAE (37 kHz, 25 °C, 15 min) increased the extraction yield by 20.5–39.7%, the protein content in APC by 2.5–12.1% and the in vitro protein digestibility by 5.6–11.03%, depending on the extraction conditions. Ultrasound treatment decreased the levels of chlorophyll and carotenoids, modified the color parameters and increased the total polyphenols and flavonoids content. Improvements in the textural, foaming and emulsifying properties of APC were also observed. UAE also reduced the scavenging capacity of 2,2-diphenyl-1-picrylhydrazyl (DPPH) radicals. However, the 2,2′-azino-bis(3-ethylbenzothiazoline-6-sulfonic acid) (ABTS•^+^) scavenging activity significantly increased in aqueous APC extracts, reaching 3118.8 mg TE/100 g DW. Overall, UAE proved effective in improving the yield and functionality of APC, supporting its application in the development of alfalfa-based protein ingredients.

## 1. Introduction

In recent years, food choices have been increasingly influenced by ethical, environmental and health considerations, leading to a growing interest in alternative sources of plant protein [1]. Alfalfa (*Medicago sativa* L.) is cultivated on approximately 2.5 million hectares in Europe, and its protein content varies between 15% and 25% of the dry matter [2,3]. Therefore, its exploitation is well justified, given its relevance not only for animal nutrition, but also as a sustainable source of protein for the food industry [4,5].

The most important product derived from alfalfa is alfalfa protein concentrate (APC), which, although traditionally used as animal feed, has recently gained increasing attention as a potential ingredient for human nutrition. APC is a heterogeneous matrix, containing 45–60% protein, essential amino acids, dietary fiber, unsaturated fatty acids, pigments, polyphenols, flavonoids, vitamins (K, B, C, D, E, folate) and minerals such as magnesium, iron, copper and potassium, among others [6,7].

To date, approximately 320 tons of APC had been consumed worldwide up to 2009, initially in India, Peru, and Congo for malnutrition alleviation, without any reported adverse effects. Since 1992, APC has been used as a dietary supplement in Mexico, Canada, and the USA, and in 2009 it also received a positive opinion from the European Food Safety Authority, with a recommended daily intake of up to 10 g [8,9].

Combined toxicological assessments, nutritional data from animal and human studies, and the long history of APC use as a dietary supplement collectively support its safety for human consumption [8,9,10,11].

Beyond its nutritional value, APC also exhibits important functional properties, including foaming capacity comparable to that of egg-white proteins, good solubility at food-relevant pH levels, and gelling ability at relatively low concentrations and temperatures across a wide pH range [12].

To produce APC, the most common method is the extraction, which consists of mechanical pressing to obtain the pressed juice, which contains approximately 35% crude protein. The juice is neutralized and preheated to coagulate the proteins, which are then recovered by centrifugation and dried under controlled conditions to preserve the bioactive compounds (BAC) [13]. A major limitation of this method is that during thermal coagulation, several undesirable components, such as pigments, volatile compounds responsible for the characteristic grassy odor, and various antinutrients, co-precipitate with the proteins. In addition, the proteins undergo thermal denaturation and exhibit reduced solubility, which may negatively affect their functional properties.

In recent years, alternative approaches for isolating APC from alfalfa juice have been developed, including isoelectric point precipitation using organic acids, endogenous fermentation [14], ultrasound, microwaves, and supercritical fluid extraction [15]; extraction has also been combined with enzymolysis in some studies [16].

Research has shown that ultrasound-assisted extraction (UAE) can significantly improve protein yield [17]. By generating cavitation and microturbulence, ultrasound effectively disrupts cell walls, promoting the rapid release of biomolecules, including associated protein fractions. This technique, which requires less time and reduces solvent consumption, not only improves extraction efficiency, but can also influence the functional properties and nutritional quality of the recovered proteins [18,19], making UAE a promising approach for the development of protein-rich ingredients for food applications [20,21].

Although UAE is considered an emerging technique for the recovery of BAC from plant materials, prolonged exposure to high-intensity ultrasound, especially at high temperatures, induces protein denaturation, impairs their functional properties, and leads to the loss of vitamins and other heat-sensitive compounds [22]. Therefore, it is crucial to evaluate how extraction methods affect the biological potential, AA, and functional characteristics of APCs. The literature indicates that only a limited number of studies have systematically characterized ultrasonically extracted APCs to ensure that the recovered proteins retain both their nutritional quality and technological functionality for applications in the food industry.

In this context, the present study was designed to explore the potential of alfalfa (*Medicago sativa* L.) as a sustainable protein source and to produce APC suitable for food applications. The study specifically examined how conventional and UAE extraction applied to frozen alfalfa aerial parts influences the extraction yield, physicochemical characteristics, functional properties including foaming, emulsification and textural attributes, color parameters, phytochemical composition and AA of APC. By systematically evaluating these factors, the research aims to provide insights into optimizing extraction strategies to improve the nutritional quality and functional performance of alfalfa-derived protein ingredients.

## 2. Materials and Methods

### 2.1. Chemicals and Plant Material

6-Hydroxy-2,5,7,8-tetramethylchromane-2-carboxylic acid (Trolox, ≥97% purity), 2,2-diphenyl-1-picrylhydrazyl hydrate (DPPH, ≥95%), and 2,2′-azino-bis(3-ethylbenzothiazoline-6-sulfonic acid) diammonium salt (ABTS, ≥98%) were obtained from Alpha Aesar (Haverhill, MA, USA). Ninhydrin reagent (≥98%), aluminum chloride hexahydrate (≥98%), and the reference standards gallic acid (GA, ≥97%) and quercetin (≥95%) were supplied by Sigma-Aldrich (St. Louis, MO, USA). The Folin–Ciocalteu phenol reagent (2.1 N) was purchased from Chem-Lab NV (Zedelgem, Belgium). Ethanol, methanol, n-hexane, sodium hydroxide, calcium oxide, lithium citrate, lithium chloride, lithium hydroxide, thiodiglycol, concentrated hydrochloric acid, anhydrous citric acid, lactic acid, concentrated nitric and perchloric acids, and trichloroacetic acid were all of analytical or chromatographic grade. Spectrophotometric measurements were carried out using a UV-1900 spectrophotometer (Shimadzu, Tokyo, Japan).

For the research, the local alfalfa variety “Dimitra” was used, which was grown in the village Talmaza, region of Stefan Voda, Republic of Moldova (46°38′09″ N 29°39′53″ E). The plants were cultivated in compliance with international legislation [23]. The plants were harvested manually, approximately 25 ± 2 cm tall, from different parts of the alfalfa plot. Before freezing, the harvested plants were cleaned, mixed for good homogeneity and vacuum-packed in 300 ± 10 g portions. The dry mater content of alfalfa was 24.03 ± 0.21%. The packaged samples were stored at −18 ± 1 °C, for 105 days.

### 2.2. Extraction of APC from Medicago sativa *L.*

**Figure 1:**
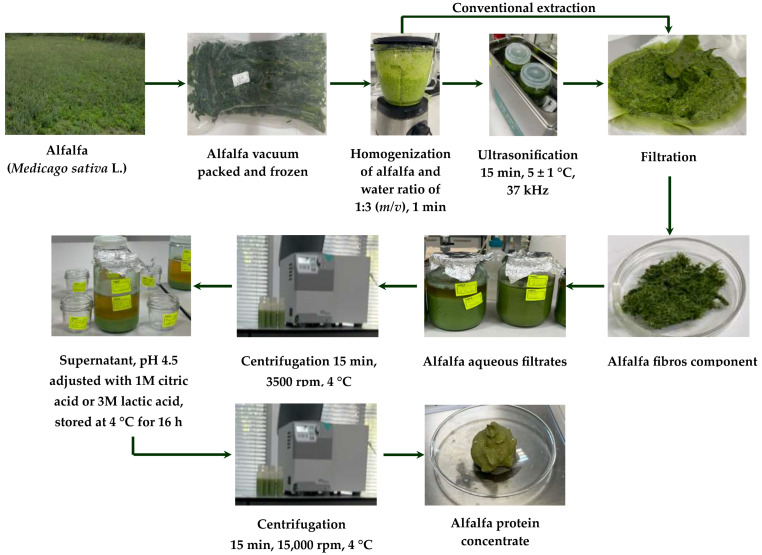
Alfalfa processing steps for APC preparation.

After 105 days of freezer storage, alfalfa aerial parts were weighed without thawing and transferred to a blender (Electrolux E6TB1-6ST, Stockholm, Sweden). Distilled water (pH 5.60 ± 0.01) or slightly alkaline water (pH 9.0 ± 0.01) was added in a sample-solvent ratio of 1:3 (*m*/*v*), the solvents maintained at 4 ± 1 °C. The mixtures were homogenized at low speed for 1 min per 100 ± 1 g of sample. The alkaline medium was prepared by adjusting the pH to 9.0 ± 0.01 using a 0.02 M Ca(OH)_2_ solution (Figure 1).

In the conventional extraction process, after mixing in a blender, the homogenates were filtered through a cotton cloth, after which the retained plant fibers were pressed by hand, and the resulting filtrates were collected.

For the UAE extraction process, after mixing in a blender, the homogenates were subjected to an additional sonication step in an ultrasonic bath (ISOLAB Laborgeräte GmbH, Eschau, Bavaria, Germany) for 15 min at 25 ± 1 °C and 37 kHz. Subsequently, the homogenates were filtered through a cotton cloth, the retained vegetable material was pressed by hand, and the filtrates were collected. The filtrates were centrifuged (15 min, 3500 rpm, 4 °C) to remove insoluble material, and the resulting supernatants were collected. Aliquots of these supernatants were then acidified with either 1 M citric acid or 3 M lactic acid to achieve a pH of 4.5, inducing isoelectric precipitation of the proteins. The samples were stored at 4 ± 1 °C for 16 h and subsequently centrifuged again to separate the proteins (15 min, 15,000 rpm, 4 °C). The resulting sediments, representing APC, were collected, weighed and the yield calculated based on dry matter content, then frozen, and stored in polystyrene containers with lids at −19 ± 1 °C. In total, eight APC samples (S1–S8) were obtained (Table 1), each prepared in triplicate, with an average dry matter content of 18.91 ± 0.8%.

### 2.3. Physicochemical Analysis

Dry matter was determined using the oven-drying technique, following the official AOAC method 925.10. The protein content (PC) was measured using the Kjeldahl method with a UDK129 unit (VELP Scientifica, Usmate, Italy). The method involves mineralizing organic nitrogen with concentrated sulfuric acid to form ammonium sulfate, followed by alkali-induced distillation of the released ammonia and its titration to determine the initial nitrogen content. A nitrogen-to-protein conversion factor of 6.25 was applied. The fat content (FC) was determined by the Soxhlet extraction method according to the AOAC official protocol 948.22, employing a SER148 solvent extraction unit (VELP Scientifica, Monza, Italy). Ash content (AC), representing the total mineral fraction, was determined by the dry incineration method. The procedure involved combusting the organic matter in a muffle furnace (OmronE5CC, Snol, Lithuania) at 550 ± 1 °C, following the AOAC 942.05 (2006) guidelines.

Water activity (a_w_) was determined using the rapid measurement method with a LabSwift-aw analyzer (Novasina AG, Lachen, Switzerland).

Titratable acidity and pH were determined using the SI Analytics pH meter, TitroLine 5000 (Weilheim, Germany), according to the method described in [24]. Titratable acidity was expressed in % lactic acid for alfalfa and in % lactic acid (Acid_lac) and % citric acid (Acid_cit) for ACP.

#### 2.3.1. Amino Acid Profile

The amino acid composition was determined by standard protocols involving cation-exchange chromatography, a method that separates amino acids according to their electrical charge. The standard reference used was SIGMA AA-S-18 [4].

#### 2.3.2. Mineral Profile

Mineral content was determined using the standard wet digestion method. Method validation parameters and detection limits followed the specifications described by Bulgaru et al. [25].

#### 2.3.3. Color Parameters

The color of the alfalfa leaves and APC samples was measured using a Chroma Meter CR-400 (Konica Minolta, Osaka, Japan) with D65 as the standard illuminant. Color values were recorded according to the CIELab color system, providing lightness (L*), red–green (a*), yellow–blue (b*) parameters and greenness (−a*/b*). High negative values of (−a*/b*) ratios indicate that alfalfa and APC samples are greener [26]. Measurements were conducted in triplicate at a controlled temperature of 21 ± 1 °C.

The total color difference (∆E*) between the APC samples and the control was calculated using method described by [27].

The chroma index (C*) represents the color intensity or saturation and is directly proportional to the vividness of the color. It was calculated according to [28].

The hue angle (h*) represents the dominant color perceived and is expressed on a 0–360° scale, where 0° corresponds to bluish-red, 90° to yellow, 180° to green, and 270° to blue. The h* value was determined by the method of [29].

### 2.4. Digestibility of Proteins

Protein digestibility was quantified as a percentage, representing the proportion of trichloroacetic acid (TCA)-soluble peptides in the supernatant generated during in vitro digestion to the total protein content of the tested samples, as described by [30].

In vitro digestion of the analyzed samples was performed according to the INFOGEST 2.0 protocol, with minor changes. The digestion process began from the second phase, the gastric phase, during which the samples were incubated under continuous agitation for 2 h at 37 °C and pH 3.0, followed by an intestinal phase lasting another 2 h at 37 °C and pH 7.0 [31]. For further analyses, the supernatant obtained after centrifugation (17,500 rpm for 10 min) was used. The concentration of TCA-soluble peptides was determined and expressed as milligrams of bovine serum albumin equivalents per kilogram of sample, following the method described in [32].

### 2.5. Analysis of Chlorophyll and Carotenoid Content

The contents of chlorophyll a (Chl a), chlorophyll b (Chl b), total chlorophyll (TChl), and carotenoid content (CC) were determined spectrophotometrically using quantification equations according to the method described by Sumanta et al. [33]. Methanolic extracts were prepared by homogenizing 0.5 g of sample with 10 mL of 99% MeOH, followed by centrifugation of the mixture (10,000 rpm, 15 min, 4 °C). The supernatant was separated, combined with an additional 4.5 mL of solvent, and analyzed spectrophotometrically by measuring absorbance at 470 nm (carotenoids), 645 nm (chlorophyll *b*), and 663 nm (chlorophyll *a*). The results were expressed in mg/100 g DW.

### 2.6. Total Polyphenols and Flavonoids

For the determination of total polyphenol content (TPC), total flavonoid content (TFC), and AA, hydroalcoholic (_alc_) and aqueous (_aq_) extracts were prepared from alfalfa and APC as follows. Ground alfalfa was extracted with either distilled water or 70% (*v*/*v*) aqueous ethanol at a sample-to-solvent ratio of 1:100 (*m*/*v*) by the UAE method at 37 kHz and 25 ± 1 °C for 15 min. APC samples were extracted with distilled water or 70% (*v*/*v*) aqueous ethanol at a ratio of 1:50 (*m*/*v*) by the UAE method under the same conditions. All extracts were centrifuged at 10,000 rpm for 10 min, and the resulting supernatants were collected for spectrophotometric analysis.

TPC and TFC in ethanolic or aqueous sample extracts were assessed spectrophotometrically following established procedures [34], with slight modifications [35]. TPC was quantified using the Folin–Ciocalteu reagent [36], based on a calibration curve prepared with gallic acid (0–500 mg/L, R^2^ = 0.9978), and results were expressed as milligrams of gallic acid equivalents per gram of dry weight (mg GAE/g DW).

TFC was measured using AlCl_3_·6H_2_O, relative to a quercetin calibration curve (0–160 mg/L, R^2^ = 0.9974), and expressed as milligrams of quercetin equivalents per gram of dry weight (mg QE/g DW).

### 2.7. Determination of Radical-Scavenging Activity

AA was evaluated for hydroalcoholic or aqueous sample solutions according to the procedure of Paulpriya et al. [37]. The DPPH radical scavenging activity was quantified using a Trolox calibration curve (0–500 µmol/L, R^2^ = 0.9994) and expressed as milligrams of Trolox equivalents per gram of dry weight (mg TE/g DW). The ABTS•^+^ radical-cation scavenging capacity was assessed using the method reported by Arnao et al. [38], and values were expressed in mg TE/g DW.

### 2.8. Foaming Capacity and Foam Stability

Foam capacity (FoC) and foam stability (FoS) of the APC samples were evaluated using the method described by Cano-Medina et al. with some modifications. APC solutions were prepared at 20 g/L, and the pH was adjusted to 4.6 using 0.01 M HCl. The solutions were vigorously mixed in graduated plastic tubes with a blender at high speed for 1 min. FoC was expressed as a percentage, calculated as the difference between the volume after agitation and the initial volume, relative to the initial volume. FoS was assessed using a similar procedure; however, the samples were left to rest for 30 min at room temperature, after which the remaining foam volume was measured. FoS was expressed as a percentage, calculated as the ratio of residual foam volume and total foam volume [39].

### 2.9. Emulsifying Activity and Emulsion Stability

The emulsifying activity (EA) and emulsion stability (ES) of APC samples were determined according to the method described by Wang and Kinsella, with slight modifications [40]. Briefly, 0.7 g of protein was dispersed in 10 mL of water, and vortexed until complete dissolution. Then, 10 mL of sunflower oil was added, and the mixture was homogenized using a Zepter mixSy (VG-022-K/VO-022-K, Baar, Switzerland) at 16,000 rpm for 1 min. The resulting oil-in-water emulsion was poured into 25 mL conical tubes and centrifuged at 3200 rpm for 5 min. The EA was expressed as a percentage, calculated as the ratio between the height of the emulsified layer and the total height of the contents in the tube. For the determination of ES, the tube was further heated in a water bath at 80 °C for 30 min, then cooled to 15 °C and centrifuged. ES was expressed as a percentage, calculated as the ratio between the height of the emulsified layer after treatment and the total height of the tube contents.

### 2.10. Texture Profile Analysis

The texture profile of the APC samples was evaluated using a Stable Micro Systems TA.HD plus C texture analyzer Surrey, UK. Measurements were conducted on cylindrical APC samples with a diameter of 30 mm, in three replicas. A double compression test was carried out with a P/5S stainless steel plate under the following conditions: pre-test speed of 1.00 mm/s, test speed of 5.00 mm/s, post-test speed of 5.00 mm/s, and a load cell capacity of 50 kg [41].

### 2.11. Statistical Analysis

The calculations in this study were performed in triplicate and are presented as mean values ± standard error of the mean. The calculations were performed using Microsoft Office Excel 2007 (Microsoft, Redmond, WA, USA). The paired *t*-tests were performed at a significance level of *p* ≤ 0.05, to determine whether the differences observed between samples treated with organic acids with and without the application of UAE were statistically significant. All experiments were performed and compared in both distilled water (pH 5.6 ± 0.01) and alkaline aqueous solution (pH 9.0 ± 0.01). These tests, Pearson correlation and Principal Component Analysis (PCA) were calculated using the Scikit-learn library (version 1.3.2) Python (version 3.10).

## 3. Results

### 3.1. Characteristics of Frozen Alfalfa

The physicochemical indices, color parameters, BAC content and AA of *Medicago sativa* L., the local alfalfa variety Dimitra (aerial parts), stored frozen at −18 ± 1 °C for 105 days, were determined (Table 2) and analyzed. PC, Table 2, in the sample of alfalfa harvested in the budding phase, was 28.63% by dry weight (DW) [42]. These values are within the limits presented by other authors, 25–32% DW for alfalfa varieties in the early stages of vegetation, when the plant has a greater amount of metabolically active leaves and a low fiber content [43,44].

The AC of 11.55% was a fairly high proportion of mineral fraction in the original alfalfa. Zhu et al. reported an average AC in alfalfa samples of 10.78% (for hay) in different regions of China [45]. The study by Luo et al. reported that alfalfa as feed material has moderate AC, although the figures vary depending on the region, soil, and contamination [46].

The FC of 2.34% (Table 2), corresponds to the expected literature data for alfalfa: in most publications and feed guides, the essential extraction for green alfalfa or hay is in the range of 1–3% DW, with values of around 1–1.5% considered typical for leaf biomass.

The acidity of alfalfa constituted 0.56% (expressed in lactic acid) and the pH value was 5.84. These values denote a weakly acidic environment for the analyzed samples, which is mainly due to the presence of organic acids specific to green plants [47]. The values obtained are within those reported by Kung et al. according to which green alfalfa presents, in the budding phase, a pH between 5.8–6.2 [48].

The a_w_ for frozen alfalfa leaves and stems was 0.748, which indicates that water is available for microbial growth. This is considered a stable a_w_ value for the food industry, but it is not high enough to control the growth of all microorganisms, as many molds and bacteria can grow at this level [49].

The in vitro digestibility of alfalfa protein was 49.37%, a relatively low value considering that part of the protein is poorly available enzymatically [50]. However, the value is within the specific limits for this plant harvested before flowering. The relatively moderate level of protein digestibility can be explained by the interactions between proteins, insoluble fibers and some antinutritional compounds (polyphenols, tannins, saponins, etc.) present in the anatomical parts of alfalfa [51].

The color of alfalfa is an important indicator of its quality, as it reflects the leaf content as well as the nutritional and biological value. In the frozen alfalfa sample, the following CIELab color parameters were determined: L*, a*, b*, C*, and h*, with values of 30.30, −21.99, 19.40, 29.32, and 138.60°, respectively (Table 2). Hu et al. [52] reported CIELab parameters for fresh alfalfa as L* = 23.04, a* = −8.98, and b* = 12.89, while Kaplan et al. [53] found a C* value of 31.65 for the same plant material. The high negative greenness value −1.13 observed in our frozen sample further supports the presence of abundant green pigments.

In the methanolic extracts obtained from alfalfa, the contents of chlorophylls *a* and *b* (Chl *a*, Chl *b*), total chlorophyll (TChl), as well as the total carotenoid content (CC), were evaluated (Table 2), and the determined values are in accordance with data obtained by other researchers. Multi-omics analyses revealed a total chlorophyll content of 12.57 mg/g at the budding stage, and 8.29 mg/g at the full-bloom stage. Chl *a* and Chl *b* contents at the budding stage: 7.45 mg/g and 4.91 mg/g, respectively; at the flowering stage: 2.82 mg/g and 1.75 mg/g, respectively [54].

The CC determined in alfalfa was 10.63 mg/100 g DW (Table 2). Values of up to 44.6 mg/kg (i.e., 4.46 mg/100 g) of β-carotene, under specific light conditions, were found for alfalfa sprouts [55].

For spectrophotometric analyses (TPC, TFC, and AA by DPPH and ABTS assays), two types of extracts-aqueous (_aq_) and hydroethanolic (_alc_), were obtained from frozen alfalfa plants. The results are presented in Table 2.

The TPC_aq_ and TFC_aq_ values for alfalfa extracts were lower (2093.6 mg GAE/100 g DW and 584.1 mg QE/100 g DW, respectively) compared to TPC_alc_ and TFC_alc_ (2583.0 mg GAE/100 g DW and 810.3 mg QE/100 g DW, respectively). This difference can be attributed to the higher efficiency of 70% aqueous ethanol in extracting polyphenolic compounds (Table 2).

The values obtained in this study were lower than those reported in the literature for methanolic extracts from alfalfa lives, which showed a TPC of 37.0 mg GAE/g DW and a TFC of 12.6 mg rutin equivalents/g DW [56], as well as 51.68 mg GAE/g and 18.55 mg QE/g, respectively [57]. At the same time, the values determined in this study are higher than the TPC and TFC reported for ethanolic extracts, which were 6.41 and 4.88 mg GAE/g DW, respectively [58].

The results show that the DPPH• radical scavenging capacity was higher in the hydroalcoholic extracts of alfalfa compared to the aqueous ones (583.2 and 485.3 mg TE/100 g DW, respectively). The AA determined by the DPPH assay is specific to phenolic compounds and depends on their concentration.

At the same time, the scavenging capacity of the ABTS•^+^ radical cation was considerably higher for the aqueous alfalfa extracts (3190.4 mg TE/100 g DW) compared to the hydroethanolic extracts (1291.9 mg TE/100 g DW).

The mineral content of *Medicago sativa* L. may vary considerably depending on the stage of growth, soil fertility, cultivar, climatic conditions, and sample preparation [44].

The data presented in Table 3 indicate a low sodium content, while potassium (29,766.3 mg/kg DW), calcium (23,025.0 mg/kg DW), magnesium (2442.3 mg/kg DW), and manganese (47.0 mg/kg DW) exhibit values consistent with those reported in the literature for the aerial parts of alfalfa at the early growth stage (K—22.1 g/kg DW, Ca—24.8 g/kg DW, Mg—3 g/kg DW, Mn—51.2 mg/kg DW). In contrast, the iron content (97 mg/kg DW) was lower than that described in previous studies (Fe—221 mg/kg DW) [44,59].

The amino acid profile of alfalfa revealed a broad composition, comprising both essential and eight non-essential amino acids (Table 4).

Among the amino acids quantified, aspartic acid (1.95 g/100 g DW) and glutamic acid (1.91 g/100 g DW) were predominant. Similar results were obtained by Luo et al. for dried alfalfa (hay) [46]. Homolka et al. showed in their study of alfalfa harvested on different days over a 30-day period that aspartic acid was the dominant amino acid, and the content of individual amino acids, as a fraction of the crude protein content, decreased with increasing plant maturity [60]. In addition, the alfalfa sample was richer in proline (1.01 g/100 g DW), leucine (0.97 g/100 g DW) and valine (0.95 g/100 g DW). Lower concentrations were observed for cysteine (0.22 g/100 g DW) and methionine (0.21 g/100 g DW). In the analyzed alfalfa samples, the essential amino acid tryptophan, known for its susceptibility to degradation, was not detected. According to literature reports, its concentration in alfalfa is generally low, approximately 0.15–0.20 mg/100 g DW [46,60]. The results obtained indicate that alfalfa contains a favorable proportion of essential amino acids that contribute to its nutritional value.

### 3.2. Influence of Extraction Method and Type of Acid Applied in Isoelectric Sedimentation on Physicochemical Indices, CIELab Color Parameters, BAC and AA of APC

#### 3.2.1. Impact of Extraction Method on the Protein Concentrates Physicochemical Indices

APC was extracted from frozen plant material using a conventional method and a UAE technique. The obtained samples were coded S1–S8 (Table 1).

The UAE method, at parameters that ensure a less destructive extraction for BAC (37 kHz, 25 °C, 15 min) [28], gave a higher yield, compared to conventional extraction. As a result, samples S2 and S4 were obtained from distilled water, with PCY of 8.76% and 7.33%, respectively, while samples S6 and S8, extracted from the alkaline medium, showed yields of 9.21% and 9.56%, respectively (Table 5). Alkalinization of the extraction solvent with Ca(OH)_2_ increases the PCY, as proteins exhibit higher solubility in an alkaline medium. The application of ultrasound facilitated the destruction of cell walls and enhanced the release of intracellular proteins [61], at the same time, unwanted enzymatic activity was reduced by directly inactivating the enzymes responsible for protein degradation [62]. Nevertheless, these recovery yields are lower compared to the crude PCY exceeding 25% DW reported by other researchers, who recovered protein from alfalfa juice obtained by direct pressing of alfalfa leaves [13,14,40].

In the UAE method, for samples extracted with distilled water, statistical analysis revealed significant differences (*p* < 0.05) between the recovery yields of proteins precipitated with lactic acid and those with citric acid, except for the insignificant values (*p* = 0.155) determined for the S1–S3 pair. On average, the recovery yield of proteins sedimented with lactic acid was more than 16% higher than that of those sedimented with citric acid. In contrast, for samples extracted in the alkaline solution adjusted with Ca(OH)_2_, both by the conventional and the UAE methods, higher yields were obtained for proteins precipitated with citric acid, which acts as a stronger chelator of Ca^2+^ ions. Thus, when citric acid is added during the pH reduction step, protein precipitation is accompanied by chelation process.

However, the PCY obtained in our experiments was lower compared to those reported in the literature. One possible reason is that the protein content of alfalfa leaves has been reported to be approximately twice that of alfalfa stems [19,63]. Another contributing factor is that the alfalfa used in this study had been frozen for 105 days, and freezing has been shown to negatively affect the yield of recovered protein [40,64].

We also assume that the hydrophilic solvent-water, extracts to a greater extent the water-soluble components from alfalfa, which represent about half of the total alfalfa proteins. According to bibliographic data, alfalfa leaves contain both hydrophobic green proteins (cell wall proteins, cell membrane proteins, leaf storage proteins, and lectins) and hydrophilic proteins, predominantly RuBisCO [65]. Alkalization of the extraction solvent with Ca(OH)_2_ was also one of the causes of the low yield. According to bibliographic data, higher protein recovery was obtained when NaOH was used, whereas protein solubility was greater when Ca(OH)_2_ served as the alkalizing agent [66].

The PC in APC is represented by high values ranging from 76.21% to 91.21%. The analyzed values are statistically significant (*p* < 0.05) for all samples S1–S8 (Table 5). The protein concentration in APC obviously depends on various factors, the most important being the vegetation period of the plant and of course the extraction methods used. The APC samples analyzed showed a higher content when using the extraction method associated with UAE, both in distilled water, a maximum relative increase of just over 9% and by 12% in alkaline water. Statistical analysis using paired *t*-tests for sample pairs S1–S2, S3–S4, S5–S6 and S7–S8 indicated that the extraction method had a significant effect (*p* < 0.05) on the protein content. Furthermore, ultrasound-assisted extraction resulted in a higher protein concentration in APC.

The results regarding the influence of pH on the PC of APC showed that in a weakly alkaline medium, proteins were extracted in slightly higher quantities compared to extraction in distilled water, by about 6–7%. The influence of the extraction medium on PC is significant (*p* < 0.05), except for S3–S7 values which are not statistically significant (*p* = 0.017). Similar results were presented by Gao et al. in the studies carried out on the obtaining of pea protein isolate [67]. Chandran et al. showed an increase in protein content with the increase in pH value, namely from 33.58% to 61.25% in more alkaline conditions [68].

The AC of APC varied within a relatively narrow range, from 0.39% (S1) to 0.53% (S8), being primarily influenced by the pH of the extraction medium, specifically by the presence of Ca(OH)_2_, and to a lesser extent by the type of acid used for isoelectric precipitation and the application of ultrasound.

Samples obtained under alkaline extraction conditions (S5–S8) exhibited significantly higher ash content (*p* < 0.05) compared to those extracted in neutral aqueous media (S1–S4) (Table 5). This phenomenon can be attributed to the formation of calcium salts of organic and inorganic acids naturally present in alfalfa during extraction, which consequently increase the ash content. Literature data also indicate that proteins solubilized using Ca(OH)_2_ retain higher calcium levels within the protein fraction [66].

The results of statistical analysis also showed that the type of acid used for isoelectric precipitation (lactic or citric) had a negligible influence on the AC (*p* > 0.05) in the samples.

The FC in the APC samples varied within a narrow range, from 1.07% to 1.23%, with no significant differences (*p* > 0.05) observed under different extraction and precipitation conditions (Table 5). Therefore, it can be concluded that the FC content in the samples was insignificantly affected by the extraction pH, the type of acid used for isoelectric precipitation (lactic or citric), or the extraction method.

The titratable acidity of the samples was calculated in relation to lactic acid (a monocarboxylic acid) and citric acid (a tricarboxylic acid) (Table 5). Comparison of the paired samples (S1–S2, S3–S4, S5–S6, and S7–S8) revealed that the UAE-extracted samples exhibited statistically significant differences (*p* < 0.05), showing higher acidity values (except for S1–S5) and corresponding pH changes, which also decreased significantly (*p* < 0.05) in UAE samples. This phenomenon can be explained by the fact that ultrasonic treatment affects the structure of plant material (e.g., enhances solvent penetration and disrupts cell walls), thereby facilitating the release of organic acids and polyphenols from the plant matrix [69]. Furthermore, the samples extracted in the alkaline medium showed lower acidity and, consequently, a lower pH (*p* < 0.05) compared to the samples extracted with distilled water.

The a_w_ of the APC samples was compared using a paired *t*-test. The analysis revealed no statistically significant differences (*p* > 0.05), indicating that the extraction conditions (medium pH, type of acid used for precipitation) and the extraction method did not affect the a_w_ of the samples. All values obtained are below the threshold of 0.80, which reflects water stability and a lower microbiological risk, but it can still be exposed to the action of molds.

The results regarding PD showed that the best results were obtained for sample S2 with 84.51% and the lowest digestibility of 70.89% for sample S5 (Table 5). Statistical analysis for all APC samples, including depending on the extraction method, extraction medium and precipitation conditions showed significant results (*p* < 0.05).

Statistical analysis of the sample pairs (S1–S2, S3–S4, S5–S6, and S7–S8) revealed a significant effect (*p* < 0.05) of the UAE extraction method on PD. Ultrasound, in addition to breaking cell walls and disaggregating protein structures, positively influences PD by opening protein molecules, making them accessible to enzymes [70]. From the results obtained, extraction with alkaline water offers higher yield but lower digestibility, samples S5–S8 have lower values compared to those obtained with distilled water. This finding is supported by the fact that high pH contributes to greater solubilization of proteins, but under these conditions, larger amounts of BAC were extracted, including antinutrients with the effect of reducing digestibility. The type of acid used for isoelectric precipitation had a lesser effect on protein digestibility. Samples S1–S4, precipitated with lactic acid, exhibited significantly higher digestibility values (*p* < 0.05) compared to those obtained using citric acid. However, the overall variation among samples remained relatively small.

#### 3.2.2. Influence of Extraction Method on CIELab Color Parameters of APC

The green color of APC will have a major influence on the quality of the food products to which it is added, thus playing a key role in shaping consumer acceptance. The CIELab color parameters were evaluated for pairs of APC samples obtained under varying extraction conditions (Table 6).

The L* ranged from 48.26 to 53.97, with significant differences observed among all pairs (*p* ≤ 0.05), except for the S7–S8 pair (*p* = 0.43). The a* parameter values varied between −13.17 and −22.80, exhibiting significant differences across all samples (*p* ≤ 0.05), except for the S5–S7 pair (*p* = 0.27). These results indicate the predominance of green pigments, primarily attributed to chlorophylls present in alfalfa. This finding further suggests that both extraction conditions and pH exert a significant influence on the green coloration of APC. The b* parameter ranged from 32.09 to 44.26, with all sample pairs showing significant differences (*p* ≤ 0.05), except for S3–S4 (*p* = 0.41), implying that the extraction conditions did not markedly affect the yellow hue associated with carotenoids in alfalfa leaves and stems. The greenness index (−a*/b*) was evaluated for all pairs of CPA samples. The results indicated that for pairs S1–S3 and S2–S4, the greenness values did not differ significantly (*p* > 0.05), suggesting that the type of organic acid used and the application of ultrasound at pH 5.6 had no substantial effect on this parameter. In contrast, the extraction conditions significantly influenced the chroma (C*) values across all compared samples (*p* ≤ 0.05). Regarding the hue angle (h*), all ACP samples exhibited values located within the third trigonometric quadrant, confirming the predominance of green coloration. However, no significant difference was observed between samples S1 and S3 (*p* = 0.63).

To assess the influence of UAE on color variation between sample pairs S1–S2, S3–S4, S5–S6, and S7–S8, the total color difference (∆E*) was calculated. The ∆E* value is a dimensionless parameter used to determine whether color differences are perceptible to the human eye, based on established sensory thresholds [27]. According to Lo Faro et al. [71], ∆E* values between 3 and 6 indicate a clearly perceptible difference, while values between 6 and 12 correspond to a strong color difference, and values greater than 12 represent completely distinct colors. In the present study, the ∆E* values for pairs S1–S2 and S3–S4 were 4.31 and 5.60, respectively, indicating perceptible color differences. The S7–S8 pair exhibited a ∆E* value of 6.75, corresponding to a pronounced difference in color, whereas the S5–S6 pair showed ∆E* > 12, signifying completely distinct coloration. These findings demonstrate that extraction under alkaline conditions significantly influenced the green coloration of the APC samples. Moreover, it has been reported that the green pigment cannot be completely removed when APCs are precipitated at their isoelectric points [19].

#### 3.2.3. Influence of Extraction Methods on Phytochemical Content and Antioxidant Activity of APC

The literature provides limited data on the total polyphenol content (TPC), total flavonoid content (TFC), and AA of APC. Samples (S1–S8) showed a light green color, indicating residual chlorophylls and chloroplastic material, and a slight grassy odor associated with volatile compounds. These components, along with carotenoids, polyphenols, vitamins, and other BACs, were retained in APC.

These compounds may interact with the protein RuBisCo, contained in alfalfa, thereby influencing its digestibility and functional and antioxidant properties. Gilani et al. reported that natural antinutritional factors present in protein concentrates, such as fiber, tannins and phytates, as well as those formed during heat or strong alkaline treatments (e.g., D-amino acids, lysinoalanine) can reduce the nutritional quality of proteins [72]. In contrast, Tanambell et al. showed that RuBisCO undergoes rapid enzymatic degradation, often within seconds, and that its digestibility is independent of processing history and purity [73].

Chl *a* and Chl *b*, TChl as well as CC contents were determined in methanol extracts from samples (S1–S8) of APC (Table 7). These values were found to be approximately 100 times lower compared to those measured in the methanolic extracts from alfalfa. The reduced pigment content in the samples is largely attributed to the hydrophilic solvent used for APC extraction in this study, as aqueous media are not suitable for solubilizing chlorophylls and carotenoids.

Statistical analysis was performed using *t*-test for the sample pairs S1–S2, S3–S4, S5–S6, and S7–S8 to evaluate the influence of the extraction method on chlorophyll and carotenoid contents. The results showed that the values recorded for samples extracted by UAE were significantly lower (*p* < 0.05) in both cases.

Carotenoids are generally resistant to heat treatments; however, they are degraded upon exposure to light and in the presence of oxidizing agents. It has been reported that ultrasound treatment promotes the isomerization, oxidation, and cleavage of *β*-carotene [74]. Analysis of the results indicates that CC is only slightly affected by the pH of the extraction solvent and the type of acid used for protein precipitation.

Statistical evaluation revealed no significant differences for the pair S1–S3 (*p* = 0.37), indicating that the type of acid used for isoelectric precipitation (lactic or acetic) had only a minor influence on the TChl of the samples. Chlorophylls are known to degrade during processing, and their stability is affected by several factors, including pH, solvent, light, oxidizing agents, temperature, and enzymatic activity. Statistical analysis performed for two groups of samples (S1, S2, S3, S4 and S5, S6, S7, S8) to evaluate the influence of pH on the TChl revealed significantly higher values (*p* < 0.05) for the samples extracted in the presence of Ca(OH)_2_.

Koca et al. reported that an alkaline environment has been used during the blanching of green vegetables to increase pH and preserve chlorophyll after processing [26]. It is well established that chlorophyll degradation occurs through its conversion into derivative compounds such as pheophytins, a process known as pheophytinization, which leads to the loss of the characteristic green color [75]. During pheophytinization, the magnesium atom in the central core of the chlorophyll molecule is replaced by two protons, resulting in the formation of pheophytin and the partial opening of the chlorophyll ring structure [76]. The removal of the Mg atom from the chlorophyll core structure is facilitated under acidic conditions, where this reaction occurs more readily [77].

TPC, TFC and AA by DPPH and ABTS, were determined by spectrophotometric analysis of two types of extracts—aqueous (_aq_) and hydroethanolic (_alc_), obtained from APC samples (S1–S8), Table 7. Statistical analysis showed that, the TPC_aq_, TFC_aq_, and DPPH_aq_ values (*p* < 0.05) were lower compared to values recorded in the hydroalcoholic extracts, except for the TPC_alc_ and TFC_alc_ in S8 (*p* > 0.05). In contrast, the aqueous extracts exhibited significantly higher ABTS values. This phenomenon can be attributed to the ability of the ABTS•^+^ radical cation to be scavenged not only by polyphenols but also by water-soluble compounds such as amino acids, peptides, and vitamins. Moreover, it has been reported that amino acids including tyrosine, tryptophan, cysteine, histidine, arginine, and cystine also contribute to the antioxidant activity detected in the ABTS decolorization assay [78,79].

Differences in TPC, TFC, and AA values between the groups of samples obtained by the conventional method (S1, S3, S5, S7) and those extracted by UAE (S2, S4, S6, S8) were assessed using *t*-test with significance set at *p* ≤ 0.05. Comparison of the paired samples revealed that the UAE-extracted APC exhibited higher polyphenol and flavonoid contents. This can be attributed to the cavitation effect generated by ultrasound, which disrupts the cellular matrix and enhances the release of BACs, proteins, and amino acids.

Comparison of DPPH_alc_ test results for the paired samples (S1–S2, S3–S4, S5–S6) showed statistically significant differences (*p* < 0.05), indicating a decrease in AA under ultrasound treatment. Similarly, in the ABTS_alc_ assay, statistically significant reductions (*p* < 0.05) were observed in the extracts following ultrasound exposure, except for the S1–S2 pair (*p* = 0.09). This phenomenon can be explained by the fact that ultrasound promotes the formation of oxidizing agents such as hydrogen peroxide and reactive oxygen species, which contribute to the degradation of sensitive BACs, including vitamins, carotenoids, and phenolic acids. Spectrophotometric analysis thus confirms the degradative effect of ultrasound on bioactive compounds soluble in 70% ethanol, resulting in a reduction of antioxidant activity.

Several studies have similarly reported that ultrasound treatment leads to the degradation of vitamins C, E, D, K, and B-complex, as well as pigments and other oxidation-sensitive compounds [28,74,80].

In contrast, the ABTS assay results for the aqueous extracts of the paired samples S3–S4 and S7–S8 (*p* < 0.05), indicating an increase in AA under ultrasound treatment, except for the S1–S2 and S5–S6 pairs (*p* > 0.05). Samples S1, S2, S5, and S6 were precipitated with lactic acid, which has been reported to induce stronger protein denaturation, thereby reducing their solubility. The observed increase in AA may be attributed to the ability of ultrasound to promote the release of proteins, peptides, amino acids, and other water-soluble compounds capable of neutralizing the ABTS•^+^ radical cation [79]. The influence of the pH of the extraction solvent on the TPC, TFC and AA values (by DPPH and ABTS) presented statistically insignificant values in the *t*-test for two groups of samples. Data (Table 7) indicated that samples S5, S6, S7, S8, extracted under mildly alkaline conditions, by both the modified conventional and UAE methods, contained higher levels of polyphenols and flavonoids (*p* < 0.05). No statistically significant differences were observed for TPC_alc_ when comparing paired samples S3–S7 (*p* = 0.30) and for TFC_alc_ in the S1–S5 pair (*p* = 0.14). Paired-sample analysis further revealed non-significant differences for S1–S5 (*p* = 0.07) and S3–S7 (*p* = 0.91), suggesting that DPPH_alc_ and DPPH_aq_ values were less affected by the pH of the extraction medium of APC. Although it was reported that alfalfa leaves contain both free, easily extractable polyphenols and cell wall-bound phenolics, the latter being released through alkaline hydrolysis [81]. It is well known that the AA of extracts largely depends on the concentration of phenolic compounds, which possess intrinsic antioxidant properties.

The organic acid, lactic or citric, applied in isoelectric sedimentation significantly influenced (*p* < 0.05) the TPC, TFC and AA values determined in the aqueous and hydroethanolic extracts (Table 7), with the APC samples precipitated with citric acid showing significantly higher values. It is likely that precipitation with lactic acid is less reversible, both for proteins and for BAC. A study on whey proteins reported lower protein resolubilization when lactic acid was used as the precipitating agent compared to citric acid, suggesting that citric acid causes less protein denaturation [82].

However, for the S5–S7 pair, the variations in TPC, TFC and AA values in the extracts were not statistically significant (*p* > 0.05). Probably, the presence of Ca^2+^ ions, which could bind some functional groups in BACs in the conventional extraction process, had a greater influence on the biological value and AA determined in samples S5 and S7.

#### 3.2.4. Influence of Extraction Methods on Textural and Functional Properties of APC

The texture properties of the APC samples, as determined in this study, are presented in Table 8. The texture was significantly influenced by the extraction method, the nature of the acid used for precipitation and the application of ultrasonic treatment. Comparing the texture parameters of samples obtained by the conventional method (S1, S3, S5, S7) with those subjected to ultrasound (S2, S4, S6, S8), an average % increase in hardness by 40–60% and a doubling of adhesiveness values were found (*p* < 0.05).

This effect can be attributed to the action of ultrasonic cavitation on the quaternary and tertiary structures of proteins, which promotes the dissociation of aggregates and the reorganization of smaller protein chains into a more compact arrangement in the resulting product. As a consequence, a denser and more stable protein network is formed, as reflected by the increased hardness and gumminess values. Arzeni et al. reported for soy protein isolate that UAE facilitates the disruption of protein aggregates and the reduction of macromolecular dimensions [83].

Statistical analysis revealed significant differences for hardness between all pairs of samples (*p* < 0.05). Similar results were reported by Gul et al., who showed that ultrasonic treatment improves the homogeneity, rigidity, and elasticity of plant protein networks [84]. Similarly, citric acid precipitated samples S4 and S8, under mildly alkaline conditions, showed higher cohesiveness (0.492–0.524) and gumminess (17.86–19.65 g) due to the ability of citric acid to chelate metal ions, promoting the association of proteins into dense aggregates. In contrast, lactic acid, being monobasic causes the formation of softer gels, due to less intense interactions between protein molecules. Statistical analysis was applied to pairs of samples S1–S3, S5–S7 to evaluate the effect of acid type on all texture parameters. The results showed significant values (*p* < 0.05) for adhesion and hardness for both pairs, and in the case of S1–S3 the differences were also significant for cohesiveness and springiness. At the same time, the differences between the values of cohesiveness and springiness were insignificant (*p* > 0.05) in the case of S5–S7.

The results obtained suggest that if the intended application involves elastic, aerated, and easily dispersible products (e.g., protein beverages, foams, bakery formulations), UAE-extracted proteins obtained under mildly alkaline conditions and precipitated with lactic acid are more suitable, as they exhibit lower gumminess and adhesivity, resulting in a softer texture that is easier to incorporate.

In contrast, for applications requiring structured or molded products (pâtés, plant-based burgers, gels, stable sauces), APC extracted via UAE under mildly alkaline conditions and precipitated with citric acid are more appropriate, due to their higher cohesiveness, increased adhesivity, and elevated gumminess, which contribute to a denser and more stable texture.

The foaming properties of proteins play a crucial role in food foaming, as they influence the texture, smoothness, and glossiness of the final product [85]. The functional properties of plant-derived proteins, considered important for manufacturers, can be affected by various factors, such as particle size, molecular structure, ionic strengths, pH, and extraction methods [84].

Overall, the ultrasound-treated samples (S2, S4, S6, S8) showed higher FoC and FoS values compared to those obtained through conventional extraction (S1, S3, S5, S7), Table 9.

The results showed that samples extracted both by conventional methods and those with the application of UAE, regarding FoC and FoS, presented significantly higher values (*p* < 0.05). For example, FoC increased from 85.19% (S1) to 88.01% (S2) and from 76.35% (S5) to 82.80% (S6), indicating that ultrasonic treatment improved the proteins’ interfacial characteristics. This enhancement is likely due to the partial unfolding of protein molecules caused by ultrasound, which exposes hydrophobic and hydrophilic groups, thereby promoting more efficient adsorption at the air–water interface, and an increase in the adhesion and flexibility of the foam resulting a stronger film formation [86]. Similar results were obtained by Gul et al. on sesame fine protein isolate [84] and Du et al. on pumpkin seed protein isolate [87].

Samples extracted under alkaline conditions showed slightly lower FoC values (73.16–82.80%) than those obtained using distilled water (84.47–88.01%), Table 9. FoS was also slightly lower than in samples extracted with distilled water, by approximately 3–4%. The data obtained are also supported by statistical processing which indicates significant values (*p* < 0.05) for samples S1–S5, S3–S7, for both FoC and FoS. This reduction can be attributed to the presence of calcium ions, which can block free carboxyl groups in compounds with foaming properties in APC. In the same context, Al-Gailani et al. established that negatively charged saponin molecules can bind to Ca^2+^ ions, forming insoluble or poorly soluble calcium-saponin complexes. Saponins are known for their ability to form stable foams in water and for exhibiting surfactant, antimicrobial and bioactive properties [88]. Regarding precipitants, lactic acid led to higher FoC and FoS than citric acid, this can be explained by less extensive aggregation during isoelectric precipitation with lactic acid. Lactic acid precipitated proteins showed significantly higher values (*p* < 0.05) for FoC of samples S1–S3 and S5–S7.

The combined use of ultrasound and lactic acid (e.g., sample S2) resulted in the most favorable foaming characteristics (FoC = 88.01%, FoS = 88.87%), confirming the synergistic effect of acoustic cavitation and controlled acidification in improving the surfactant behavior of APC. The obtained data was also supported by the results of the statistical analysis, *t*-test.

The emulsifying activity, defined as the ability to disperse oil droplets within an aqueous phase under mechanical force, ranged from 43.1 to 57.5%, while the emulsion stability ranged from 40.2 to 62.2% (Table 9). These values are within the bibliographic data reported for alfalfa proteins [40] and indicate that the protein concentrates (S1–S8) exhibit surface-active properties similar to those of other food proteins [39]. The formed emulsions were relatively stable, in samples S2, S4, and S6-S8, more than half of the emulsified layer remained stable after heating, cooling, and centrifugation treatments.

The results indicate significantly higher (*p* < 0.05) values of EA and ES in the samples extracted under alkaline conditions, which can be attributed to the presence of very low concentrations of calcium ions, a phenomenon explained by other researchers [89]. Ye et al. determined that a low dose of Ca(OH)_2_/Ca^2+^ can improve emulsification by compacting/stabilizing the protein film at the interface, and higher concentrations cause ionic bonding, flocculation/aggregation, and phase separation [90].

Statistical analysis was applied to the sample pairs S1–S2, S3–S4, S5–S6, and S7–S8 to evaluate the effect of the extraction method on EA and ES. The results showed that the samples extracted by ultrasounds exhibited significantly higher values (*p* < 0.05) in both cases. The EA of the UAE extracted samples (S2 and S4) increased markedly, by more than 14%, compared to their corresponding conventionally extracted counterparts (S1 and S3). Similarly, ES showed a substantial increase in the UAE-extracts, by 31.8% in S2, 24.5% in S4, 27.3% in S6, and 14.2% in S8.

Similar observations were made by O’Sullivan et al., who reported that ultrasound treatment enhanced the emulsifying performance of both animal and plant proteins compared to untreated counterparts. Ultrasound treatment was reported to reduce protein size and hydrodynamic volume while modifying molecular structure. For instance, emulsions prepared from ultrasound-treated pea protein isolates exhibited smaller droplet sizes compared to those made with untreated proteins, which correlates with reduced interfacial tension between the two systems [91].

### 3.3. Relationships Between Physicochemical Characteristics, CIELab Color Parameters, Biologically Active Compounds, Texture Profile, and Functional Properties in APC

**Figure 2:**
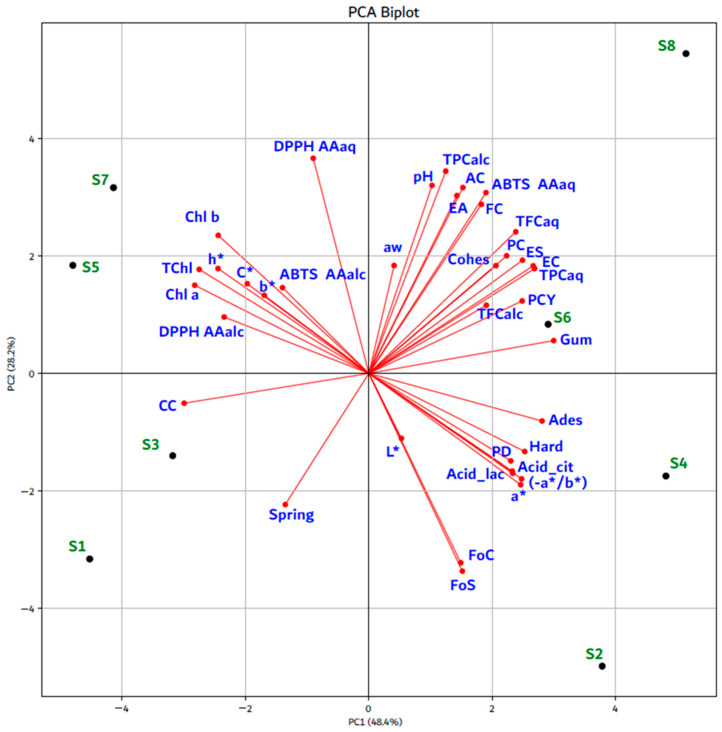
Principal component analysis: S1 and S3—alfalfa protein concentrates extracted with distilled water, (pH 5.6 ± 0.01), followed by isoelectric precipitation with lactic and citric acid respectively; S2 and S4—alfalfa protein concentrates extracted by UAE (15 min) in distilled water (pH 5.6 ± 0.01), followed by isoelectric precipitation with lactic and citric acid respectively; S5 and S7—alfalfa protein concentrates extracted with alkaline aqueous solution (pH 9.0 ± 0.01), followed by isoelectric precipitation with lactic and citric acid respectively; S6 and S8—alfalfa protein concentrates extracted by UAE (15 min) in alkaline aqueous solution (pH 9.0 ± 0.01), followed by isoelectric precipitation with lactic and citric acid respectively; PCY—alfalfa protein concentrate yield; PC—protein content; AC—ash content; FC—fat content; Acid_cit—titratable acidity, expressed in citric acid; Acid_lac—titratable acidity, expressed in lactic acid; a_w_—water activity; PD—protein digestibility; L*—lightness; a*—red–green parameter; b*—yellow−blue parameter; (−a*/b*)—greenness; C*—chroma index; h*—hue angle; Chl *a*—chlorophyll *a*; Chl *b*—chlorophyll *b*; TChl—total chlorophylls; CC—carotenoid content; TPC_aq_ and TPC_alc_—total polyphenol content in aqueous and hydroethanolic extracts; TFC_aq_ and TFC_alc_—total flavonoid content in aqueous and hydroethanolic extracts; ABTS—2,2-azino-bis-3-ethylbenzothiazoline-6-sulphonic acid; ABTS AA_aq_ and AA_alc_—antioxidant activity in aqueous and hydroethanolic extracts; DPPH—2,2-diphenyl-1-picrylhydrazyl; DPPH AA_aq_ and AA_alc_—antioxidant activity in aqueous and hydroethanolic extracts; Hard—hardness; Ades—adesivity; Spring—springines; Cohes—cohesiviness; Gum—gumminess; FoC—foaming capacity; FoS—foaming stability; EA—emulsifying activity; ES—emulsion stability.

Principal Component Analysis (PCA) was used to illustrate the relationships between physicochemical characteristics, CIELab color parameters, BACs, texture profile analysis, and functional properties in APC, Figure 2. The first two principal components, PC1 and PC2, accounted for 48.4% and 28.2% of the total variance. The results highlight that several parameters are closely associated, showing highly significant correlations (*p* < 0.05) (Figure S1). For example, DPPH AA_alc_ was strongly correlated with Chl *a* (r = 0.82), TChl (r = 0.78), CC (r = 0.73) and Chl *b* (r = 0.65). Similarly, ABTS AA_aq_ was strongly correlated with TFC_aq_ (r = 0.96), TPC_aq_ (r = 0.91), PC (r = 0.76). h* was closely related to Chl *a* (r = 0.94), TChl (r = 0.93), Chl *b* (r = 0.86) and CC (r = 0.73). In addition, PC correlated with ES (r = 0.94), EC (r = 0.87), Gum (r = 0.75), Cohes (r = 0.70); FC and ES (r = 0.84), EC (r = 0.81), Cohes (r = 0.91), Gum (r = 0.71) and (−a*/b*) showed negative correlations with Chl *a* (r = −0.95), TChl (r = −0.94), Chl *b* (r = −0.87) and CC (r = −0.74) (Figure S1). PC1 was mainly associated with Gum, PCY, TFC_alc_, EC, ES, PC, Cohes, TFC_aq_, DPPH AA_alc_, Chl *a*, TChl, h*, b*, C*, Chl *b*, ABTS AA_alc_, TPC_aq_, CC, Ades, Hard, PD, Acid_cit, (−a*/b*), a* and Acid_lac, whereas PC2 was primarily related to DPPH AA_aq_, a_w_, pH, TPC_alc_, EA, AC, FC, ABTS AA_aq_, L*, FoS, FoC, and Spring. According to the PCA plot, the extraction conditions (pH 5.6 or 9.0) and the type of isoelectric precipitation (lactic or citric acid) influenced the samples distribution. Under these conditions, samples S1 and S3 were located in the lower left region of the plot, whereas S5 and S7 appeared in the upper left region. When ultrasound was applied under the same conditions, samples S2 and S4 were grouped in the lower right region, while S6 and S8 were positioned at the upper right. Both principal components differentiated S1 and S3 from S2 and S4, as well as S5 and S7 from S6 and S8, indicating an inverse relationship between extraction conditions (pH 5.6 or 9.0), the type of isoelectric precipitation, and the presence or absence of ultrasound. Specifically, PC1 distinguished S6 and S8 from S4 and S2, and S1 and S3 from S5 and S7. PC2 differentiated S6 and S8 from S5 and S7, and S1 and S3 from S2 and S4. Regarding the correlations of APC with the analyzed characteristics, in our opinion it seems that S1 and S3 are rather related to CC and Spring; S2 and S4 to L*, FoS, FoC, Acid_lac, PD, a*, Acid_cit, Hard, Ades and (−a*/b*); S6 and S8 to Gum, TFC_alc_, PCY, TPC_aq_, EC, ES, PC, Cohes, TFC_aq_, FC, ABTS AA_aq_, FC, EA, AC, TPC_alc_, pH and a_w_; S5 and S7 to DPPH AA_alc_, DPPH AA_aq_, Chl *a*, TChl, TChl *b*, b*, C*, h* and ABTS AA_alc_.

## 4. Limitations of the Study

During the extraction of APC, a fibrous by-product rich in minerals, proteins, BACs and amino acids is generated, and its characteristics warrant further investigation. Such residues can be exploited as a source of phytonutrients or as an ingredient for animal feed.

Given the scope of this study further research is needed to comprehensively evaluate the mineral composition of the obtained samples, including the presence of lead and other heavy metals, as well as to characterize their amino acid profiles and functional properties, such as emulsification capacity.

Future work should also clarify how variations in extraction conditions, such as wider solvent pH ranges and solid:liquid ratios or the use of alternative organic acids, influence the yield, physicochemical properties, phytochemical content, functional proprieties, et al. of APC.

It is also necessary to apply advanced physicochemical methods, for example High-Performance Liquid Chromatography, Gas Chromatography, Mass-spectrometry, to describe the qualitative phytochemical profile of APC.

## 5. Conclusions

Frozen alfalfa was investigated to evaluate its potential as a source of alfalfa protein concentrates (APC) for food applications. Amino acid analysis of alfalfa aerial parts revealed eight essential amino acids, with tryptophan being undetectable due to its sensitivity to processing and analytical methods. Paired *t*-tests (*p* ≤ 0.05) showed that the applied ultrasound regime significantly increased the extraction yield and in vitro protein digestibility and improved the functional properties of APC, especially under slightly alkaline conditions (presence of Ca(OH)_2_) with citric acid precipitation. The UAE method influenced the color parameters by reducing the pigment level, increasing the total polyphenol and flavonoid content, decreasing the DPPH radical scavenging activity, but increasing the AA via the ABTS test in aqueous APC extracts. In addition, UAE concentrates obtained under alkaline conditions and precipitated with lactic acid presented textural properties characterized by lower gumminess and adhesion, compared to those precipitated isoelectrically with citric acid, which presented higher cohesion, adhesion and gumminess. Also, the action of ultrasound improved the FoC, FoS, EC, and EA of APC.

## 6. Future Outlook

The incorporation of alfalfa (*Medicago sativa* L.) protein concentrate in the food industry presents considerable potential owing to its balanced nutritional composition and valuable functional properties. Future investigations should aim to optimize extraction and purification processes to improve both protein yield and functional performance. Additionally, the inclusion of alfalfa proteins in plant-based formulations may contribute to the diversification of protein sources and the reduction of reliance on conventional crops. Advances in bioprocessing, enzymatic modification, and microencapsulation technologies are expected to enhance the sensory attributes and digestibility of alfalfa proteins, thereby facilitating their application in a broad spectrum of food products. Overall, alfalfa protein concentrate constitutes a promising ingredient for the formulation of sustainable and functionally enhanced foods that align with the increasing global demand for plant-based alternatives.

## Figures and Tables

**Table 1:** Naming and coding of APC samples.

Solvent	Acid of Isoelectric Sedimentation	Conventional Extraction	Ultrasound-Assisted Extraction (15 min)
Distilled water, pH 5.6 ± 0.01	Lactic acid	S1	S2
	Citric acid	S3	S4
Alkaline aqueous solution, pH 9.0 ± 0.01	Lactic acid	S5	S6
	Citric acid	S7	S8

**Table 2:** Physicochemical indices, BAC, AA and CIELab color parameters of the frozen *Medicago sativa* L. used for experiments (the results are expressed as means ± standard deviations of three experiments).

Indices	Quantity
**Physicochemical Indices**	
PC, %	28.63 ± 0.71
AC, %	11.55 ± 0.11
FC, %	2.34 ± 0.40
Titratable acidity, % expressed in lactic acid	0.56 ± 0.013
pH	5.84 ± 0.01
a_w_, c.u.	0.748 ± 0.001
PD, %	49.37 ± 0.39
**CIELab color parameters**	
L*	30.30 ± 0.29
a*	−21.99 ± 0.32
b*	19.40 ± 0.53
−a*/b*	−1.13 ± 0.42
C*	29.32 ± 0.62
h*, °	138.60 ± 0.45
**Biologically active compounds**	
Chl *a*, mg/100 g DW	1315.9 ± 2.2
Chl *b*, mg/100 g DW	452.8 ± 1.9
TChl, mg/100 g DW	1768.7 ± 2.0
CC, mg/100 g DW	10.63 ± 1.7
TPC_aq_, mg GAE/100 g DW	2093.6 ± 7.5 ^a^
TPC_alc_, mg GAE/100 g DW	2583.0 ± 4.9 ^b^
TFC_aq_, mg QE/100 g DW	584.1 ± 1.1 ^a^
TFC_alc_, mg QE/100 g DW	810.3 ± 1.3 ^b^
ABTS AA_aq_, mg TE/100 g DW	3190.4 ± 1.7 ^b^
ABTS AA_alc_, mg TE/100 g DW	1291.9 ± 4.0 ^a^
DPPH AA_aq_, mg TE/100 g DW	485.3 ± 1.2 ^a^
DPPH AA_alc_, mg TE/100 g DW	583.2 ± 0.7 ^b^

PC—protein content; AC—ash content; FC—fat content; a_w_—water activity; PD—protein digestibility; L*—lightness; a*—red–green parameter; b*—yellow–blue parameter; −a*/b*—greenness; C*—chroma index; h*—hue angle; Chl *a*—chlorophylls *a*; Chl *b*—chlorophylls *b*; TChl—total chlorophylls; CC—carotenoid content; TPC_aq_ and TPC_alc_—total polyphenol content in aqueous and hydroethanolic extracts; TFC_aq_ and TFC_alc_—total flavonoid content in aqueous and hydroethanolic extracts; ABTS—2,2-azino-bis-3-ethylbenzothiazoline-6-sulphonic acid; ABTS AA_aq_ and AA_alc_—antioxidant activity in aqueous and hydroethanolic extracts; DPPH—2,2-diphenyl-1-picrylhydrazyl; DPPH AA_aq_ and AA_alc_—antioxidant activity in aqueous and hydroethanolic extracts; GAE—gallic acid equivalents; QE—quercetin equivalents; TE—trolox equivalents; DW—dry weight; different letters (^a,b^) in the following rows (TPC_aq_ and TPC_alc_; TFC_aq_ and TFC_alc_; ABTS AA_aq_ and AA_alc_; DPPH AA_aq_ and AA_alc_) indicate significant differences (*p* ≤ 0.05) related to the influence of the extraction solvent.

**Table 3:** Mineral composition of the frozen *Medicago sativa* L. used for experiments (the results are expressed as means ± standard deviations of three experiments).

Alfalfa (*Medicago sativa* L.)	Mineral Content, mg/kg DW					
	Sodium	Potassium	Magnesium	Calcium	Manganese	Iron
	217.5 ± 9.6	29,766.3 ± 46	2442.3 ± 21	23,025.0 ± 37	47.0 ± 1.8	97.0 ± 3.2

**Table 4:** Amino acid compositions of the frozen *Medicago sativa* L. used for experiments (the results are expressed as means ± standard deviations of three experiments).

Amino Acids, g/100 g DW	Value
Aspartic acid	1.95 ± 0.06
Threonine *	0.64 ± 0.02
Serine	0.74 ± 0.01
Glutamic *	1.91 ± 0.08
Proline	1.01 ± 0.06
Glycine	0.84 ± 0.04
Alanine	0.75 ± 0.04
Valine *	0.95 ± 0.05
Cysteine	0.22 ± 0.01
Methionine *	0.21 ± 0.01
Isoleucine *	0.75 ± 0.05
Leucine *	0.97 ± 0.08
Tyrosine	0.64 ± 0.06
Phenylalanine *	0.84 ± 0.07
Lysine	0.95 ± 0.08
Histidine *	0.63 ± 0.06
Arginine	0.87 ± 0.09
∑FAAs	14.93 ± 0.86
∑INM	1.95 ± 0.14
∑NEAAs	8.05 ± 0.61
∑EAAs	6.88 ± 0.52
∑IAAs	4.58 ± 0.09
∑GAAs	13.01 ± 0.75
∑AAsK	2.23 ± 0.05
∑AAsP	14.93 ± 0.86

*—essential amino acids; ∑FAAs—the total free amino acids; ∑INM—indicators of nitrogen metabolism; ∑NEAAs—non-essential amino acids; ∑EAAs—essential amino acids; ∑IAAs—immunoactive amino acids; ∑GAAs—glycogen amino acids; ∑AAsK—amino acids ketogenic; ∑AAsP—amino acids proteinogenic.

**Table 5:** Influence of extraction method and type of acid applied in isoelectric sedimentation on APC physicochemical indices (the results are expressed as means ± standard deviations of three experiments).

Physicochemical Indices	Samples							
	S1	S2	S3	S4	S5	S6	S7	S8
PCY, %	6.27 ± 0.25 ^a^	8.76 ± 0.25 ^e^	6.08 ± 0.20 ^a^	7.33 ± 0.31 ^c^	6.75 ± 0.24 ^b^	9.21 ± 0.35 ^e^	7.14 ± 0.26 ^d^	9.56 ± 0.39 ^f^
PC, %	76.21 ± 0.60 ^a^	83.45 ± 0.87 ^c^	83.52 ± 0.53 ^d^	85.61 ± 0.77 ^f^	77.32 ± 0.81 ^b^	86.67 ± 0.67 ^g^	85.51 ± 0.60 ^e^	91.21 ± 0.77 ^h^
AC, %	0.39 ± 0.02 ^a^	0.44 ± 0.01 ^b^	0.42 ± 0.02 ^a^	0.46 ± 0.03 ^c^	0.47 ± 0.02 ^d^	0.51 ± 0.04 ^d^	0.48 ± 0.02 ^d^	0.53 ± 0.05 ^d^
FC, %	1.07 ± 0.05 ^a^	1.08 ± 0.07 ^a^	1.12 ± 0.08 ^a^	1.17 ± 0.18 ^a^	1.10 ± 0.06 ^a^	1.15 ± 0.10 ^a^	1.14 ± 0.09 ^a^	1.23 ± 0.19 ^a^
Titratable acidity, % expressed in lactic acid	1.15 ± 0.02 ^c^	1.40 ± 0.01 ^g^	1.26 ± 0.02 ^e^	1.57 ± 0.02 ^h^	1.10 ± 0.01 ^a^	1.22 ± 0.01 ^d^	1.12 ± 0.01 ^b^	1.27 ± 0.01 ^f^
Titratable acidity, % expressed in citric acid	0.81 ± 0.01 ^a^	1.03 ± 0.01 ^f^	0.89 ± 0.01 ^d^	1.12 ± 0.01 ^g^	0.78 ± 0.01 ^a^	0.86 ± 0.01 ^c^	0.83 ± 0.03 ^b^	0.91 ± 0.02 ^e^
pH, c.u.	4.24 ± 0.02 ^c^	4.15 ± 0.02 ^a^	4.29 ± 0.02 ^d^	4.16 ± 0.01 ^b^	4.50 ± 0.01 ^g^	4.37 ± 0.02 ^f^	4.53 ± 0.01 ^h^	4.31 ± 0.02 ^e^
a_w_, c.u.	0.775 ± 0.001 ^c^	0.770 ± 0.001 ^a^	0.772 ± 0.001 ^d^	0.773 ± 0.002 ^d^	0.773 ± 0.002 ^d^	0.773 ± 0.001 ^d^	0.771 ± 0.001 ^b^	0.777 ± 0.002 ^e^
PD, %	78.15 ± 0.49 ^e^	84.51 ± 0.32 ^h^	74.26 ± 0.34 ^c^	80.72 ± 0.47 ^g^	70.89 ± 0.44 ^a^	78.71 ± 0.48 ^f^	73.49 ± 0.22 ^b^	77.57 ± 0.20 ^d^

PCY—protein concentrate yield; PC—protein content; AC—ash content; FC—fat content; a_w_—water activity; PD—protein digestibility; S1 and S3—alfalfa protein concentrates extracted with distilled water, (pH 5.6 ± 0.01), followed by isoelectric precipitation with lactic and citric acid respectively; S2 and S4—alfalfa protein concentrates extracted by UAE (15 min) in distilled water (pH 5.6 ± 0.01), followed by isoelectric precipitation with lactic and citric acid respectively; S5 and S7—alfalfa protein concentrates extracted with alkaline aqueous solution (pH 9.0 ± 0.01), followed by isoelectric precipitation with lactic and citric acid respectively; S6 and S8—alfalfa protein concentrates extracted by UAE (15 min) in alkaline aqueous solution (pH 9.0 ± 0.01), followed by isoelectric precipitation with lactic and citric acid respectively. Different letters (^a–h^) in each row indicate significant differences (*p* ≤ 0.05). Paired *t*-tests were performed at a significance level of *p* ≤ 0.05 to determine whether the observed differences between samples existed.

**Table 6:** Influence of extraction method and type of acid applied in isoelectric sedimentation on CIELab color parameters of APC (the results are expressed as means ± standard deviations of three experiments).

Indices	Samples							
	S1	S2	S3	S4	S5	S6	S7	S8
L*	52.30 ± 0.23 ^d^	53.97 ± 0.18 ^g^	48.26 ± 0.29 ^a^	52.21 ± 0.24 ^e^	53.09 ± 0.39 ^f^	50.26 ± 0.28 ^b^	51.02 ± 0.23 ^c^	51.39 ± 0.45 ^c^
a*	−19.04 ± 0.22 ^f^	−15.07 ± 0.23 ^b^	−17.14 ± 0.22 ^d^	−13.17 ± 0.33 ^a^	−22.27 ± 0.35 ^g^	−15.15 ± 0.22 ^c^	−22.80 ± 0.19 ^g^	−17.55 ± 0.32 ^e^
b*	36.12 ± 0.39 ^d^	36.24 ± 0.45 ^d^	32.24 ± 0.41 ^b^	32.14 ± 0.35 ^b^	44.26 ± 0.49 ^f^	32.09 ± 0.25 ^a^	40.05 ± 0.18 ^e^	35.83 ± 0.24 ^c^
−a*/b*	−0.53 ± 0.01 ^e^	−0.41 ± 0.01 ^a^	−0.53 ± 0.01 ^e^	−0.41 ± 0.01 ^a^	−0.50 ± 0.01 ^c^	−0.47 ± 0.01 ^d^	−0.57 ± 0.01 ^f^	−0.49 ± 0.01 ^b^
C*	40.83 ± 0.22 ^f^	39.25 ± 0.51 ^d^	36.51 ± 0.49 ^c^	34.73 ± 0.47 ^a^	49.55 ± 0.56 ^h^	35.49 ± 0.34 ^b^	46.08 ± 0.26 ^g^	39.90 ± 0.36 ^e^
h*, **°**	117.8 ± 0.6 ^f^	112.6 ± 0.2 ^b^	118.0 ± 0.1 ^f^	112.3 ± 0.4 ^a^	116.7 ± 0.2 ^e^	115.3 ± 0.2 ^c^	119.6 ± 0.2 ^g^	116.1 ± 0.4 ^d^
ΔE*	-	4.31 ± 0.51	-	5.60 ± 0.23	-	14.38 ± 0.26	-	6.75 ± 0.11
Sample images	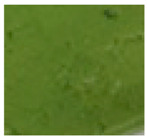	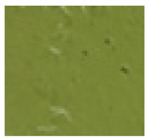	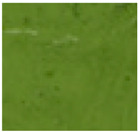	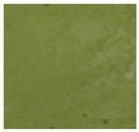	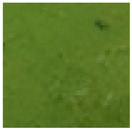	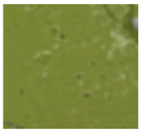	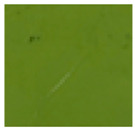	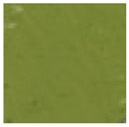

L*—lightness; a*—red–green parameter; b*—yellow–blue parameter; −a*/b*—greenness; C*—chroma index; h*—hue angle; ΔE*—total color difference (between S1–S2, S3–S4, S5–S6, S7–S8); S1 and S3—alfalfa protein concentrates extracted with distilled water, (pH 5.6 ± 0.01), followed by isoelectric precipitation with lactic and citric acid respectively; S2 and S4—alfalfa protein concentrates extracted by UAE (15 min) in distilled water (pH 5.6 ± 0.01), followed by isoelectric precipitation with lactic and citric acid respectively; S5 and S7—alfalfa protein concentrates extracted with alkaline aqueous solution (pH 9.0 ± 0.01), followed by isoelectric precipitation with lactic and citric acid respectively; S6 and S8—alfalfa protein concentrates extracted by UAE (15 min) in alkaline aqueous solution (pH 9.0 ± 0.01), followed by isoelectric precipitation with lactic and citric acid respectively. Different letters (^a–h^) in each row indicate significant differences (*p* ≤ 0.05). Paired *t*-tests were performed at a significance level of *p* ≤ 0.05 to determine whether the observed differences between samples existed.

**Table 7:** Influence of extraction method and type of acid applied in isoelectric sedimentation on BAC and AA of APC (the results are expressed as means ± standard deviations of three experiments).

Indices	Samples							
	S1	S2	S3	S4	S5	S6	S7	S8
Chl *a*, mg/100 g DW	12.73 ± 0.75 ^e^	3.67 ± 0.58 ^a^	11.91 ± 0.59 ^e^	4.25 ± 0.55 ^b^	13.57 ± 0.78 ^e^	7.52 ± 0.83^c^	14.25 ± 0.58 ^f^	8.09 ± 0.81 ^d^
Chl *b*, mg/100 g DW	4.03 ± 0.25 ^e^	1.40 ± 0.66 ^a^	4.10 ± 0.40 ^e^	1.83 ± 0.25 ^b^	6.08 ± 0.88 ^f^	3.64 ± 0.51 ^d^	6.10 ± 0.50 ^f^	3.53 ± 0.45 ^c^
TChl, mg/100 g DW	16.76 ± 0.72 ^e^	5.07 ± 0.45 ^a^	16.01 ± 0.43 ^e^	6.08 ± 0.10 ^b^	19.65 ± 0.23 ^f^	11.16 ± 0.14 ^c^	20.35 ± 0.07 ^g^	11.62 ± 0.14 ^d^
CC, mg/100 g DW	3.07 ± 0.21 ^h^	0.60 ± 0.20 ^b^	2.93 ± 0.15 ^g^	0.67 ± 0.06 ^c^	2.83 ± 0.15 ^f^	1.13 ± 0.06 ^d^	2.40 ± 0.10 ^e^	0.09 ± 0.10 ^a^
TPC_aq_, mg GAE/100 g DW	1347.3 ± 6.6 ^a^	1965.0 ± 6.0 ^d^	1355.7 ± 4.3 ^a^	2553.8 ± 8.4 ^f^	1589.8 ± 5.6 ^b^	2237.3 ± 6.3 ^e^	1680.7 ± 4.3 ^c^	3118.8 ± 10.9 ^g^
TFC_aq_, mg QE/100 g DW	261.54 ± 5.27 ^b^	428.28 ± 1.20 ^d^	241.79 ± 2.71 ^a^	693.77 ± 1.91 ^g^	395.05 ± 1.55 ^c^	649.27 ± 1.78 ^f^	487.40 ± 1.30 ^e^	945.29 ± 1.12 ^h^
ABTS AA_aq_, mg TE/100 g DW	1699.3 ± 5.3 ^a^	1771.5 ± 5.6 ^a,b,c^	1709.9 ± 3.3 ^a,b^	1911.4 ± 4.6 ^e^	1849.3 ± 3.7 ^a,b,c,d^	1934.0 ± 6.8 ^d^	1860.6 ± 3.4 ^d^	2194.9 ± 6.8 ^f^
DPPH AA_aq_, mg TE/100 g DW	397.16 ± 6.81 ^a^	360.68 ± 2.04 ^a^	406.31 ± 3.73 ^a^	382.56 ± 4.31 ^a^	417.18 ± 4.59 ^a,b^	414.37 ± 5.41 ^b^	421.97 ± 2.49 ^b^	430.70 ± 3.11 ^b^
TPC_alc_, mg GAE/100 g DW	2107.2 ± 7.11 ^a^	2250.4 ± 3.8 ^b^	2488.7 ± 7.6 ^c^	2585.3 ± 6.3 ^c,d,e^	2663.9 ± 2.6 ^f^	2669.0 ± 4.1 ^f^	2535.2 ± 3.7 ^c,d,e,f^	3045.3 ± 5.1 ^g^
TFC_alc_, mg QE/100 g DW	782.36 ± 2.21 ^b^	795.33 ± 3.11 ^d^	784.72 ± 2.76 ^b,c^	798.96 ± 2.03 ^e^	756.78 ± 5.78 ^a^	759.98 ± 4.32 ^a^	738.03 ± 3.16 ^a^	901.69 ± 2.87 ^f^
ABTS AA_alc_, mg TE/100 g DW	1304.4 ± 3.3 ^b^	1259.6 ± 4.2 ^b^	1447.1 ± 2.4 ^e,f^	1393.7 ± 4.6 ^d^	1462.5 ± 4.4 ^g^	1201.0 ± 2.2 ^a^	1417.0 ± 3.4 ^e^	1376.6 ± 2.7 ^c^
DPPH AA_alc_, mg TE/100 g DW	607.34 ± 1.5 ^a,b^	523.60 ± 1.61 ^a^	605.23 ± 1.41 ^a,b^	558.14 ± 1.82 ^b^	588.0 ± 2.06 ^a,b^	515.10 ± 1.61 ^a,b^	608.98 ± 3.67 ^a,b^	573.73 ± 2.01 ^b^

Chl *a*—chlorophyll *a*; Chl *b*—chlorophyll *b*; TChl—total chlorophylls; CC—carotenoid content; TPC_aq_ and TPC_alc_—total polyphenol content in aqueous and hydroethanolic extracts; TFC_aq_ and TFC_alc_—total flavonoid content in aqueous and hydroethanolic extracts; ABTS—2,2-azino-bis-3-ethylbenzothiazoline-6-sulphonic acid; ABTS AA_aq_ and AA_alc_—antioxidant activity in aqueous and hydroethanolic extracts; DPPH—2,2-diphenyl-1-picrylhydrazyl; DPPH AA_aq_ and AA_alc_—antioxidant activity in aqueous and hydroethanolic extracts; GAE—gallic acid equivalents; QE—quercetin equivalents; TE—trolox equivalents; DW—dry weight. S1 and S3—alfalfa protein concentrates extracted with distilled water, (pH 5.6 ± 0.01), followed by isoelectric precipitation with lactic and citric acid respectively; S2 and S4—alfalfa protein concentrates extracted by UAE (15 min) in distilled water (pH 5.6 ± 0.01), followed by isoelectric precipitation with lactic and citric acid respectively; S5 and S7—alfalfa protein concentrates extracted with alkaline aqueous solution (pH 9.0 ± 0.01), followed by isoelectric precipitation with lactic and citric acid respectively; S6 and S8—alfalfa protein concentrates extracted by UAE (15 min) in alkaline aqueous solution (pH 9.0 ± 0.01), followed by isoelectric precipitation with lactic and citric acid respectively. Different letters (^a–h^) in each row indicate significant differences (*p* ≤ 0.05). Paired *t*-tests were performed at a significance level of *p* ≤ 0.05 to determine whether the observed differences between samples existed.

**Table 8:** Texture parameters of APC samples.

Indices	Samples							
	S1	S2	S3	S4	S5	S6	S7	S8
Hardness, g	29.76 ± 0.70 ^c^	49.19 ± 0.37 ^g^	28.19 ± 0.23 ^c^	36.32 ± 0.51 ^d^	27.15 ± 0.69 ^b^	43.66 ± 0.60 ^f^	25.50 ± 0.73 ^a^	37.90 ± 0.23 ^e^
Adhesivity, g‧s	16.69 ± 0.59 ^b^	67.40 ± 0.76 ^h^	22.38 ± 0.68 ^d^	47.39 ± 0.65 ^f^	13.22 ± 0.73 ^a^	36.33 ± 0.39 ^e^	18.56 ± 0.40 ^c^	53.59 ± 0.51 ^g^
Springiness, %	1.022 ± 0.003 ^e^	0.999 ± 0.002 ^c^	0.995 ± 0.003 ^a^	1.000 ± 0.002 ^c^	0.999 ± 0.003 ^c^	1.001 ± 0.002 ^c^	0.999 ± 0.003 ^a,b,c^	0.998 ± 0.002 ^b^
Cohesiveness, %	0.319 ± 0.014 ^a^	0.305 ± 0.005 ^a^	0.354 ± 0.021 ^d^	0.492 ± 0.012 ^f^	0.323 ± 0.011 ^a,b^	0.363 ± 0.021 ^e^	0.333 ± 0.019 ^b,c,d^	0.524 ± 0.013 ^g^
Gumminess, g	9.57 ± 0.52 ^c^	14.91 ± 0.25 ^d^	9.87 ± 0.33 ^c^	17.86 ± 0.51 ^f^	8.62 ± 0.41 ^a^	15.67 ± 0.40 ^e^	8.95 ± 0.56 ^a,b^	19.65 ± 0.51 ^g^

S1 and S3—alfalfa protein concentrates extracted with distilled water, (pH 5.6 ± 0.01), followed by isoelectric precipitation with lactic and citric acid respectively; S2 and S4—alfalfa protein concentrates extracted by UAE (15 min) in distilled water (pH 5.6 ± 0.01), followed by isoelectric precipitation with lactic and citric acid respectively; S5 and S7—alfalfa protein concentrates extracted with alkaline aqueous solution (pH 9.0 ± 0.01), followed by isoelectric precipitation with lactic and citric acid respectively; S6 and S8—alfalfa protein concentrates extracted by UAE (15 min) in alkaline aqueous solution (pH 9.0 ± 0.01), followed by isoelectric precipitation with lactic and citric acid respectively. Different letters (^a^^–^^h^) in each row indicate significant differences (*p* ≤ 0.05). Paired *t*-tests were performed at a significance level of *p* ≤ 0.05 to determine whether the observed differences between samples existed.

**Table 9:** Functional Properties of APC.

Indices	Samples							
	S1	S2	S3	S4	S5	S6	S7	S8
FoC, %	85.19 ± 0.43 ^f^	88.01 ± 0.55 ^h^	84.47 ± 0.28 ^d^	87.88 ± 0.36 ^g^	76.35 ± 0.30 ^b^	82.80 ± 0.26 ^e^	73.16 ± 0.40 ^a^	79.64 ± 0.33 ^c^
FoS, %	86.30 ± 0.30 ^f^	88.87 ± 0.30 ^h^	84.97 ± 0.20 ^d^	87.05 ± 0.30 ^g^	83.97 ± 0.19 ^c^	85.92 ± 0.32 ^e^	81.81 ± 0.18 ^a^	83.92 ± 0.19 ^b^
EA, %	43.09 ± 0.21 ^a^	49.34 ± 0.24 ^c^	43.70 ± 0.27 ^b^	50.84 ± 0.17 ^d^	52.39 ± 0.35 ^e^	55.80 ± 0.27 ^g^	54.50 ± 0.32 ^f^	57.51 ± 0.27 ^h^
ES, %	40.19 ± 0.77 ^a^	52.72 ± 0.96 ^d^	45.80 ± 0.47 ^c^	57.03 ± 0.67 ^g^	44.37 ± 0.70 ^b^	56.04 ± 0.61 ^f^	54.46 ± 0.17 ^e^	62.17 ± 0.34 ^h^

FoC—foam capacity; FoS—foam stability; EA—emulsifying activity; ES—emulsion stability. S1 and S3—alfalfa protein concentrates extracted with distilled water, (pH 5.6 ± 0.01), followed by isoelectric precipitation with lactic and citric acid respectively; S2 and S4—alfalfa protein concentrates extracted by UAE (15 min) in distilled water (pH 5.6 ± 0.01), followed by isoelectric precipitation with lactic and citric acid respectively; S5 and S7—alfalfa protein concentrates extracted with alkaline aqueous solution (pH 9.0 ± 0.01), followed by isoelectric precipitation with lactic and citric acid respectively; S6 and S8—alfalfa protein concentrates extracted by UAE (15 min) in alkaline aqueous solution (pH 9.0 ± 0.01), followed by isoelectric precipitation with lactic and citric acid respectively. Different letters (^a–h^) in each row indicate significant differences (*p* ≤ 0.05). Paired *t*-tests were performed at a significance level of *p* ≤ 0.05 to determine whether the observed differences between samples existed.

## Data Availability

The original contributions presented in the study are included in the article/Supplementary Materials.

## Supplementary Materials

The following supporting information can be downloaded at: https://www.mdpi.com/article/10.3390/foods14244309/s1, Figure S1: The correlation values between physicochemical characteristics, CIELab color parameters, biologically active compounds, texture profile analysis, and functional properties in APC.
