# Supplementary material for: Effect of Conventional and Ultrasound-Assisted Extraction Conditions on the Physicochemical Properties, Phytochemical Content, Antioxidant Activity and Functional Properties of Alfalfa Protein Concentrates
Source: Foods. 2025 Dec 14;14(24):4309. doi: 10.3390/foods14244309 (PMC12733140; doi:10.3390/foods14244309)
Supplement: Supplementary file 1 [file foods-14-04309-s001.zip › foods-3995958-supplementary.pdf]

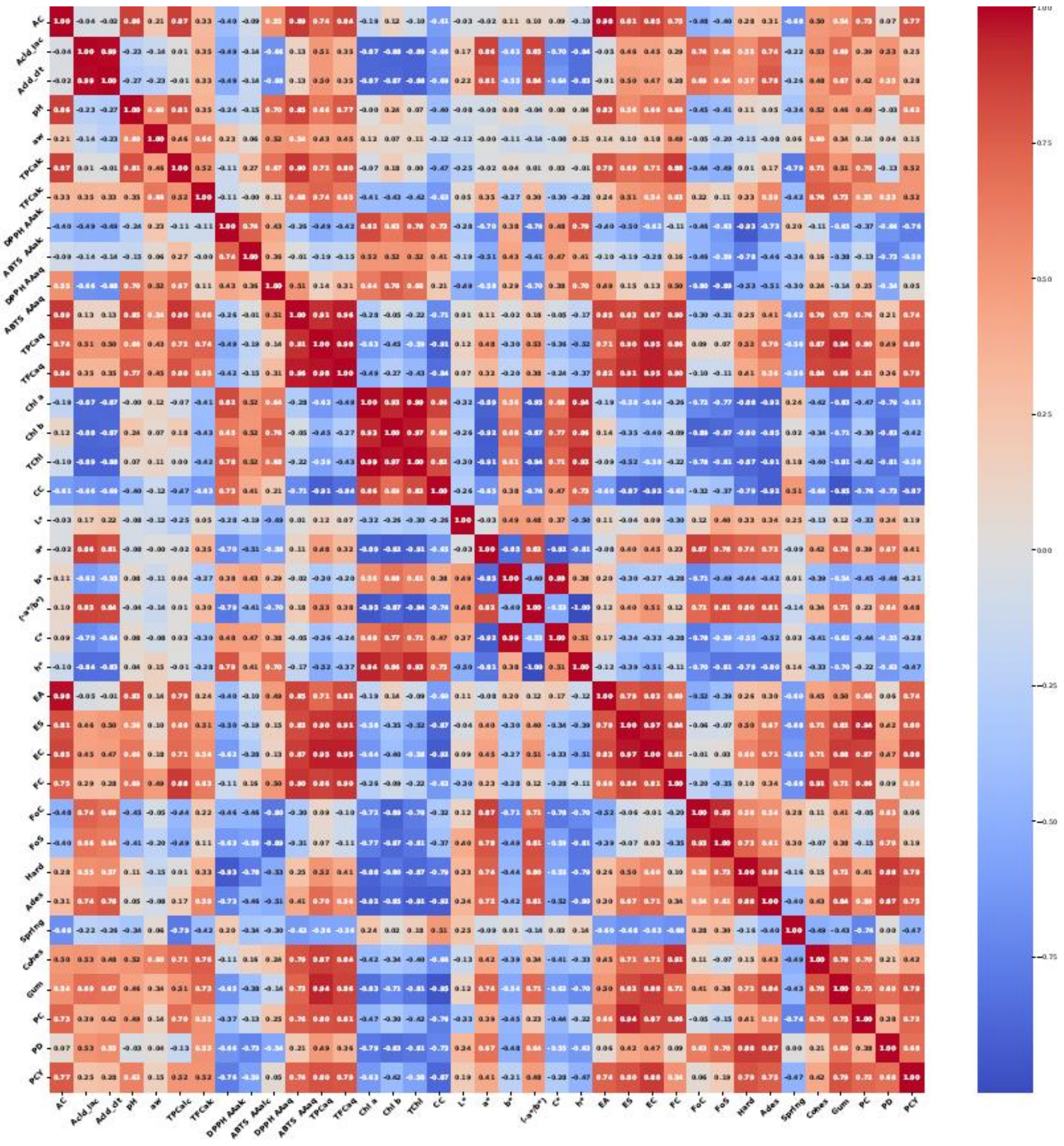

Figure S1. The correlation values between physicochemical characteristics, CIELab color parameters, biologically active compounds, texture profile analysis, and functional properties in APC: S1 and S3 - alfalfa protein concentrates extracted with distilled water, (pH 5.6 ± 0.01), followed by isoelectric precipitation with lactic and citric acid respectively; S2 and S4 - alfalfa protein concentrates extracted by UAE (15 min) in distilled water (pH 5.6 ± 0.01), followed by isoelectric precipitation with lactic and citric acid respectively; S5 and S7 - alfalfa protein concentrates extracted with alkaline aqueous solution (pH 9.0 ± 0.01), followed by isoelectric precipitation with lactic and citric acid respectively; S6 and S8 - alfalfa protein concentrates extracted by UAE (15 min) in alkaline aqueous solution (pH 9.0 ± 0.01), followed by isoelectric precipitation with lactic and citric acid respectively; PCY- alfalfa protein concentrate yield; PC - protein content; AC – ash content; FC – fat content; Acid_cit – titratable acidity, expressed in citric acid; Acid_lac – titratable acidity, expressed in lactic acid; a_w - water activity; PD- protein digestibility; L* - lightness; a* - red–green parameter; b* - yellow–blue parameter; (-a*/b*) - greenness; C* - chroma index; h* - hue angle; Chl *a* - chlorophyll *a*; Chl *b* - chlorophyll *b*; TChl - total chlorophylls; CC - carotenoid content; TPC_{aq} and TPC_{alc} – total polyphenol content in aqueous and hydroethanolic extracts; TFC_{aq} and TFC_{alc} – total flavonoid content in aqueous and hydroethanolic extracts; ABTS - 2,2-azino-bis-3-ethylbenzothiazoline-6-sulphonic acid; ABTS AA_{aq} and AA_{alc} – antioxidant activity in aqueous and hydroethanolic extracts; DPPH - 2,2-diphenyl-1-picrylhydrazyl; DPPH AA_{aq} and AA_{alc} – antioxidant activity in aqueous and hydroethanolic extracts; Hard – hardness; Ades – adesivity; Spring – springines; Cohes – cohesiviness; Gum – gumminess; FoC – foaming capacity; FoS – foaming stability; EA – emulsifying activity; ES – emulsion stability.
